# Phenolic compounds, antioxidant capacity, and α‐amylase and α‐glucosidase inhibitory activity of ethanol extracts of perilla seed meal

**DOI:** 10.1002/fsn3.3419

**Published:** 2023-07-05

**Authors:** Ga‐Young Choi, Young‐Sil Han, Ki‐Hyeon Sim, Myung‐Hyun Kim

**Affiliations:** ^1^ Department of Food & Nutrition Sookmyung Women's University Seoul South Korea; ^2^ Major in Traditional Culinary Culture, Graduate School of Arts Sookmyung Women's University Seoul South Korea; ^3^ Department of Culinary Arts Traditional Korean Cuisine Major Baewha Women's University Seoul South Korea

**Keywords:** antioxidant activity, by‐product, enzyme inhibitory activity, perilla seed meal, phenolic compound

## Abstract

*Perilla frutescens* is a medicinal herb that is commonly cultivated in Asian countries. Perilla seed is extensively pressed for cooking oil extraction. However, phenolic chemicals are still abundant in pressed perilla seed meal (PSM), which was previously thought to be useless after oil extraction. In our study, PSM was extracted using five solvents (water and 25%, 50%, 75%, and 100% ethanol) based on different ethanol concentrations, and its antioxidant activity, phenolic compounds, and inhibitory effects against key enzymes related to diabetes mellitus were evaluated. The 75% ethanol extract had higher phenolic (105.58 mg GAE/g DW) and flavonoid (66.52 mg QE/g DW) contents and showed better antioxidant and inhibitory effects against α‐glucosidase and α‐amylase. Analysis of the phenolic compounds of the five extracts by HPLC indicated the presence of apigenin, rosmarinic acid, benzoic acid, caffeic acid, and vanillic acid. Therefore, because of its high antioxidant activity and inhibitory capacity against enzymes relevant to diabetes, the 75% ethanol extract of perilla seed meal has the most potential to be used as a functional or nutraceutical food in the prevention and treatment of oxidation and diabetes.

## Graphical abstract

Because the potential value and application of perilla seed meal lack supporting scientific data, we also investigated the effects of ethanol solvents on antioxidant activity, inhibitory effects against key enzymes relevant to diabetes, and the extraction of polyphenol from perilla seed meal by in vitro methods.

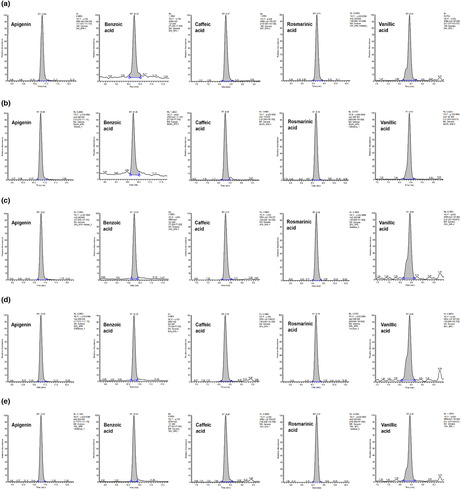

## 1 INTRODUCTION

Perilla (*Perilla frutescens* (L.) Britt.) is an annual plant and member of the mint family, *Lamiaceae* (Ha et al., 2012). Perilla seed is widely cultivated in Asian countries such as South Korea, China, Japan, and India. Perilla leaves are used in salads, sushi, garnish, soup, and herbs to treat depression‐related diseases and asthma, and the seeds are mainly consumed following oil extraction. Perilla seed has been demonstrated to exert antioxidant, antiglycosuria, antiallergic, antimicrobial, antitumor, and anticancer activities due to containing phenolic compounds, rosmarinic acid, unsaturated fatty acids, essential oil, and vitamins (Ha et al., 2012, Jun et al., 2014). Perilla seed is high in polyphenols and includes many unsaturated fatty acids like α‐linolenic acid. Moreover, perilla seed contains over 90% unsaturated fatty acids and its α‐linolenic acid content ranges from 52.58% to 61.98% (Youfang et al., 2012). The contents of these phenolic compounds vary significantly according to the extraction solvent. Consequently, polar solvents are frequently used for recovering polyphenols from plant matrices. Additionally, ethanol is known as a good solvent for polyphenol extraction and is safe for human consumption (Dai & Mumper, 2010).

Perilla oil inhibits the formation of a chemical mediator in various allergic and inflammatory reactions; it also has antibacterial and anticancer properties and can lower blood pressure, alleviate thrombosis, and inhibit cancer cell growth (Asif, 2011). Although perilla seed has a high unsaturated fatty acid content, many other compounds have been identified from the leaves and seeds, such as the flavonoids apigenin and luteolin (Lee et al., 2013), and phenolic compounds, such as caffeic acid and rosmarinic acid (Ha et al., 2012).

However, the by‐product of *P. frutescent* seed, called perilla seed meal (PSM), following oil extraction is usually used as animal feed or discarded as waste. To our knowledge, although many of the compounds exhibit potential antitumor effects, which are attributed to their significant antioxidant activities, few reports have examined the phenolic compound components in PSM. Because PSM could be a potential resource of natural phenolic antioxidants, we examined the phenolic compounds in PSM and compared their contents, antioxidant activities, and inhibitory effects against enzymes relevant to diabetes. Because the potential value and application of PSM lack supporting scientific data, we also investigated the effects of ethanol solvents on antioxidant activity, inhibitory effects against key enzymes relevant to diabetes, and the extraction of polyphenol from PSM by in vitro methods.

## 2 MATERIALS AND METHODS

### 2.1 Sample

The flesh perilla seed meal (PSM) cultivated in Gwangju‐si, Gyeonggi‐do, South Korea, in June 2021, was used as the sample in this experiment. The perilla seed was stir fried at 120°C for 15 min. The PSM was produced after immediate oil extraction, then ground and stored at −55°C during the experiment.

### 2.2 Preparation of perilla seed meal extracts (PSME)

The extraction of PSM was performed using the method of Bernaert et al. (2012) with modification. The PSM was mixed with each of the water and ethanol solvents (25%, 50%, 75%, and 100%) with a ratio of 1:10, three times at a 3 h interval in water at 70°C. The perilla seed meal extracts (PSME), the water extract (WE), 25% ethanol extract (25EE), 50% ethanol extract (50EE), 75% ethanol extract (75EE), and 100% ethanol extract (100EE) were filtered and evaporated under vacuum at 70°C until the volume of the solution left was 10 mL. These extracts were freeze‐dried for 72 h.

### 2.3 Standards and reagents

Folin–Ciocalteu phenol reagent, DPPH (2,2‐diphenyl‐1‐picrylhydrazyl), ABTS (2,2′‐azino‐bis (3‐ethylbenzothiazoline‐6‐sulfonate acid)), α‐amylase, α‐glucosidase, Trolox, 4‐N‐trophenyl‐α‐D‐glucopyranoside (*p*NPG), vitamin C, apigenin, benzoic acid, caffeic acid, kaempferol, rosmarinic acid, vanillic acid, gallic acid, and quercetin were purchased from Sigma Chemical Co. Acetonitrile, trichloroacetic acid, potassium chloride, sodium acetate, sodium carbonate (Na_2_CO_3_), sodium hydroxide (NaOH), sodium phosphate, and ferric chloride were purchased from Duksan Reagents.

### 2.4 Determination of total polyphenol content (TPC)

The total polyphenol content of PSME was determined based on the method of Folin–Ciocalteu by Swain and Hillis (1959). Briefly, samples (150 μL) dissolved in distilled water were mixed with 2 N Folin–Ciocalteu reagent (50 μL) and these mixtures were reacted at room temperature for 3 min. Then, after 800 μL of 5% Na_2_CO_3_ was added, the mixtures were reacted in darkness for 2 h. The absorbance was measured using a UV/VS spectrophotometer (T‐60, PG Instruments) at 725 nm. TPC results were expressed as mg gallic acid equivalents (GAE)/g dry weight (DW). Each sample was analyzed in triplicate.

### 2.5 Determination of total flavonoid content (TFC)

The total flavonoid content of PSME was measured based on the method by Davis (1947) with slight modification. Briefly, samples (1 mL) dissolved in distilled water were mixed with 90% diethylene glycol (10 mL). The mixtures were added with 1 N NaOH (1 mL) and reacted at 37°C for 1 h. The absorbance was measured at 420 nm. TFC results were expressed as mg quercetin equivalents (QE)/g dry weight (DW). Each sample was analyzed in triplicate.

### 2.6 Oxygen radical absorbance capacity (ORAC) assay

The oxygen radical absorbing capacity (ORAC) assay was carried out by Bernaert et al.'s (2012) method. The assay was performed in 10 mM phosphate buffer (pH 7.4) with a final reaction volume of 200 μL and assayed using a fluorescent microplate reader (SpectraMax i3x Multi‐Mode, Molecular Devices). Briefly, samples (25 μL) and 10 nM fluorescein sodium (150 μL) were placed in wells of the 96‐well microplate, and then preincubated at 37°C for 30 min. After incubation, 240 mM AAPH solution (25 μL) was quickly added to the mixtures. The microplate was immediately placed in the reader, and fluorescence intensity values of each well were detected every 90 s for 2 h at an excitation wavelength of 485 nm and an emission wavelength of 520 nm. The phosphate buffer (25 μL) was used instead of the sample as a blank. The ORAC value was calculated using the equation below:

AUC=1+f1/f0+f2/f0+f3/f0+…+f80/f

where *f*0 is the initial fluorescence reading. Trolox was used as the standard. The ORAC value was reported as μM Trolox equivalents (TE)/g of dry weight (DW). Each sample was analyzed in triplicate.

### 2.7 DPPH and ABTS radical scavenging activity assay

The DPPH radical scavenging activity assay was performed by the method of Blois (1958) with some modifications. The samples (3 mL) were mixed with 1 mL of DPPH solution (1.5 × 10^−4^ M, in ethanol). The mixtures were incubated in darkness for 30 min at room temperature and measured at 517 nm to evaluate the absorbance using a UV/VIS spectrophotometer.

The ABTS radical scavenging activity assay was performed by the method of Re et al. (1999) with some modifications. The ABTS^+^ solution was dissolved in distilled water to acquire a final concentration of 7 mM, then mixed with 2.45 mM potassium persulfate (1:1, v/v). The mixtures were incubated in darkness for 12–16 h at room temperature until the reaction was completed. The mixtures were diluted with PBS buffer to an absorbance of 0.70 ± 0.02 at 734 nm. The samples (100 μL) of different concentrations were added to ABTS working solution (900 μL). The mixtures were incubated for 3 min at 25°C in the dark, and the absorbance of the mixtures was measured at 734 nm using a UV/VIS spectrophotometer. The radical scavenging activity was calculated using the equation below:

$$\text{Radical scavenging activity}\;\left(\%\right)=\left(1-A_{\text{sample}}/A_{\text{control}}\right)\times 100$$

The ability of extracts to scavenge activity was expressed using the half‐maximal inhibitory concentration (IC_50_) value. Each sample was analyzed in triplicate.

### 2.8 Reducing power assay

The reducing power assay of the PSME was tested by the method of Oyaizu (1986) with some modifications. At first, samples (1 mL) of the same concentration were added to the test tubes containing 1 mL of 0.2 M sodium phosphate buffer (pH 6.6) and 1 mL of 1% potassium ferricyanide, and the mixture was incubated for 20 min at 50°C. Then, 10% trichloroacetic acid (1 mL) was added to the mixture and centrifuged for 10 min at 3000 rpm. Finally, the supernatant (1 mL) was added to the mixture containing distilled water (1 mL) and 0.1% ferric chloride (0.2 mL). After 10 min reaction, the absorbance of the mixtures was measured at 700 nm using a UV/VIS spectrophotometer. L‐ascorbic acid and Trolox were used as a standard. Each sample was analyzed in triplicate.

### 2.9 α‐Amylase and α‐glucosidase inhibitory assay

The α‐amylase inhibitory activity was determined by the method of Bhandari et al. (2008) with slight modification. Starch azure (2 mg), which was used as a substrate, was mixed in 1 mL of 0.5 M Tris–HCl buffer (pH 6.9) containing 0.01 M CaCl_2_. The starch azure was boiled for 5 min, and then preincubated at 37°C for 5 min. Samples (200 μL) diluted in distilled water, 200 μL of 1 U/mL α‐amylase solution was mixed, and the starch azure solution (300 μL) was added to the mixture. Sample test tubes were incubated at 37°C for 10 min and the reaction was stopped by adding 100 μL of 50% acetic acid. The reacted mixture was centrifuged (3000 rpm, 4°C) for 10 min. The absorbance was measured at 595 nm.

The α‐glucosidase inhibitory activity was carried out according to the method of Xu et al. (2018) with some modifications. Briefly, samples diluted in distilled water at various concentrations were mixed with 10 μL of 1 U/mL α‐glucosidase solution (in 0.05 M sodium phosphate buffer, pH 6.8). The mixture was incubated at 37°C for 5 min. The 200 μL of 1 mM pNPG solution was added to the mixture. The mixture was reacted at 37°C for 20 min, and then the reaction was stopped by adding 1 N NaOH solution (500 μL). The 0.05 M sodium phosphate buffer (590 μL) was added to the mixture for a final reaction volume of 1500 μL. The absorbance was measured at 405 nm. Acarbose was used as a standard. α‐Amylase and α‐glucosidase inhibitory capacities were calculated according to the following formula:

$$\text{Inhibitory rate}\;\left(\%\right)=\left[1-\left(A_{\text{sample}}-A_{\text{sample blank}}\right)/A_{\text{control}}\right]\times 100$$

The ability of extracts to enzyme inhibitory activity was expressed using the half‐maximal inhibitory concentration (IC_50_) value. Each sample was analyzed in triplicate.

### 2.10 LC–MS/MS analysis

Phenolic compounds of PSME were determined by high‐performance liquid chromatography using standard chemicals such as apigenin, benzoic acid, caffeic acid, kaempferol, rosmarinic acid, and vanillic acid. Standards and samples were diluted in ethanol and distilled water of various concentrations, respectively. The analysis was performed using a triple quadrupole LC–MS spectrometry (Finnigan TSQ Quantum Ultra EMR, Thermo Scientific) equipped with an electrospray ionization (ESI) and negative mode. Column: ROC C18 (3.0 × 150 mm, 5 μm, Restekm); flow rate: 200 μL/min; injection volume: 10 μL; column temperature: 30°C; mobile phase: 0.1% acetic acid in water (A) and 0.1% acetic acid in acetonitrile (B) gradient elution (0–2 min: A 90%; 12–14 min: A 0%; and 15–20 min: A 90%); mass spectrometer spray voltage: 3000 V, sheath gas pressure: 40; auxiliary gas pressure: 10; and capillary temperature: 270°C. The analysis of extracts was conducted by comparing the obtained molecular ions and fragmentation patterns of LC–MS/MS results with data from the literature and with a mass library for the standard compounds.

### 2.11 Statistical analysis

All experimental results were analyzed using the SPSS program (Statistical Analysis Program, version 25, IBM Co.). One‐way analysis of variance (ANOVA) and Duncan's multiple‐range test were used at the level of significance of *p* < .05. The data were expressed as mean ± standard deviation (SD). Microsoft Excel software (Microsoft) was used to generate graphics.

## 3 RESULTS AND DISCUSSION

### 3.1 Total phenolic and flavonoid contents

Previous studies indicated that the perilla seed meal (PSM) antioxidant was strongly and positively correlated with the existence of phenolic and flavonoid contents. Polyphenols, which are important plant secondary metabolites, have potential benefits for humans (Rahman et al., 2018). The major phenolic contents of PSM, such as rosmarinic acid, are important contributors to its antioxidant activity (Zhang et al., 2017). Phenolic and flavonoid contents in the perilla seed meal extracts (PSME; Table 1) differed significantly (*p* < .001). The total phenolic contents in the water extract (WE), 25% ethanol extract (25EE), 50% ethanol extract (50EE), 75% ethanol extract (75EE), and 100% ethanol extract (100EE) were 73.84 ± 0.49, 75.81 ± 0.99, 85.43 ± 3.69, 105.58 ± 4.11, and 35.27 ± 2.56 mg GAE/g, respectively. The total flavonoid contents in the WE, 25EE, 50EE, 75EE, and 100EE were 33.91 ± 0.31, 36.63 ± 1.19, 48.12 ± 1.90, 66.52 ± 3.02, and 23.55 ± 0.98 mg QE/g, respectively. The total phenolic and flavonoid contents increased from the WE to the 75EE. However, the contents decreased in the 100EE. The highest value of total phenolic and flavonoid contents was obtained from the 75EE and the lowest value was found in the 100EE. Hung et al. (2021) reported *Camellia sinensis* twig extracts with the highest total phenolic and flavonoid contents were obtained from extraction with 50% ethanol as compared to extracts with 0%, 10%, and 95% ethanol. In addition, the total phenolic and flavonoid contents increased from 0% to 50% ethanol extracts but dropped for the 95% ethanol extract. The antioxidant activity in the extracts depends on several factors, such as solvent polarity, pH, extraction temperature, time, and sample composition (Gullon et al., 2017). Karabegović et al. (2018) indicated the total phenolic and flavonoid contents in *Solanum retroflexum* Dun. fruit were the highest in the 75% ethanol extract as compared to the other tested ethanol extracts.

**TABLE 1:** Total phenolic and flavonoid contents and oxygen radical absorbance capacity (ORAC) of perilla seed meal of ethanol extract with different concentrations.

Solvents	Total phenolic contents (mg GAE/g)	Total flavonoid contents (mg QE/g)	ORAC (μM TE/mg)
Water extract	73.84 ± 0.49^c^	33.91 ± 0.31^c^	1150.22 ± 33.86^d^
25% ethanol extract	75.81 ± 0.99^c^	36.63 ± 1.19^c^	1389.46 ± 63.02^c^
50% ethanol extract	85.43 ± 3.69^b^	48.12 ± 1.90^b^	1536.20 ± 96.44^b^
75% ethanol extract	105.58 ± 4.11^a^	66.52 ± 3.02^a^	2727.94 ± 65.27^a^
100% ethanol extract	35.27 ± 2.56^d^	23.55 ± 0.98^d^	271.40 ± 10.88^e^

*Note*: Means followed by different letters (a–e) within the same column are significantly different (*p* < .05). All values were expressed as mean ± SD (*n* = 3).

Abbreviations: GAE, gallic acid equivalent; QE, quercetin equivalent; TE, Trolox equivalent.

### 3.2 Oxygen radical absorbance capacity (ORAC)

As shown in Table 1, the oxygen radical absorbance capacity in the WE, 25EE, 50EE, 75EE, and 100EE were 1150.22 ± 33.86, 1389.46 ± 63.02, 1536.20 ± 96.44, 2727.94 ± 65.27, and 271.40 ± 10.88 μM TE/mg, respectively (*p* < .001). Moreover, the 75EE had the highest positive effect on the ORAC value. Thus, antioxidant compounds appear to be more extractable in the 75EE than in other extracts. Al Jitan et al. (2018) indicated that the 75% ethanol extracts of *Tetraena qatarensis* and *Arthrocnemum macrostachyum* showed excellent antioxidant activity as compared to the 25EE and 50EE.

### 3.3 DPPH and ABTS radical scavenging activity

**FIGURE 1:**
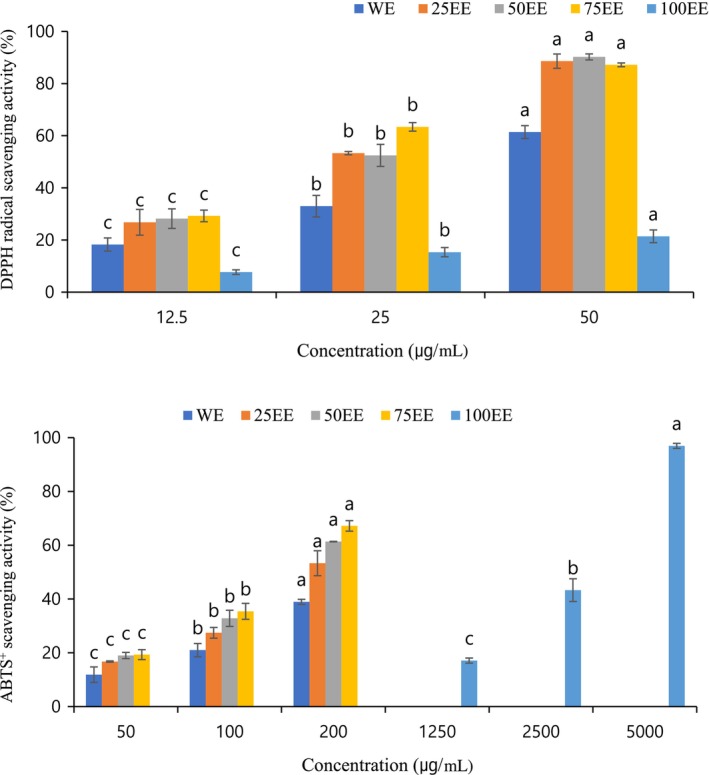
2,2‐diphenyl‐1‐picrylhydrazyl (DPPH) and ABTS^+^ scavenging activity of perilla seed meal of ethanol extract with different concentrations, water extract (WE), 25% ethanol extract (25EE), 50% ethanol extract (50EE), 75% ethanol extract (75EE), and 100% ethanol extract (100EE). Values with different letters (a–c) in different concentrations are significantly different (*p* < .05).

The 2,2‐diphenyl‐1‐picrylhydrazyl (DPPH) and 2,2′‐azino‐bis (3‐ethylbenzothiazoline‐6‐sulfonic acid) (ABTS) assays were used to evaluate antioxidant potential based on the ability to scavenge nonbiological stable‐free radicals. From the IC_50_ values (Table 2), the 75EE was able to interact with DPPH efficiently and quickly because the IC_50_ value was lower than that of other extracts, which had IC_50_ values of 20.10 ± 0.54 (*p* < .001). The DPPH radical scavenging activity of the 75EE was also significantly higher than the other extracts at all concentrations. In addition, a concentration‐dependent pattern was found in the DPPH scavenging assay of the ethanol extracts (Figure 1). Karabegović et al. (2018) reported that the highest DPPH radical scavenging activity was obtained from extraction with 75% ethanol as compared to the 25% and 50% ethanol extractions. In the ABTS experiment, a similar pattern was observed. With a scavenging ability of 146.18 ± 5.94, the 75EE outperformed the other extracts, and the scavenging capacity percentage of the ABTS radical was the highest (*p* < .001). In addition, the L‐ascorbic acid and Trolox antioxidant capacities on the DPPH and ABTS scavenging tests in our study were better than that of the PSME. Wang et al. (2016) indicated that the ABTS or DPPH scavenging radical ability was correlated with the concentration and polymerization degree of organ antioxidants.

**TABLE 2:** 2,2‐diphenyl‐1‐picrylhydrazyl (DPPH) and 2,2′‐azino‐bis (3‐ethylbenzothiazoline‐6‐sulfonate acid) (ABTS) radical scavenging activity of perilla seed meal of ethanol extract with different concentrations.

Solvents	DPPH radical scavenging activity IC_50_ (μg/mL)	ABTS radical scavenging activity IC_50_ (μg/mL)
Water extract	40.10 ± 2.29^b^	266.08 ± 4.89^b^
25% ethanol extract	23.23 ± 0.57^c^	187.79 ± 1.03^c^
50% ethanol extract	25.00 ± 2.80^c^	160.16 ± 3.32^c^
75% ethanol extract	20.10 ± 0.54^c^	146.18 ± 5.94^c^
100% ethanol extract	214.39 ± 17.64^a^	2766.57 ± 88.25^a^
L‐ascorbic acid	1.77 ± 0.03^d^	24.29 ± 0.34^d^
Trolox	2.19 ± 0.12^d^	29.61 ± 1.73^d^

*Note*: Means followed by different letters (a–d) within the same column are significantly different (*p* < .05). All values were expressed as mean ± SD (*n* = 3). The IC_50_ values are defined as the extract concentration to inhibit 50% of free radicals under assayed conditions.

### 3.4 Reducing power

**FIGURE 2:**
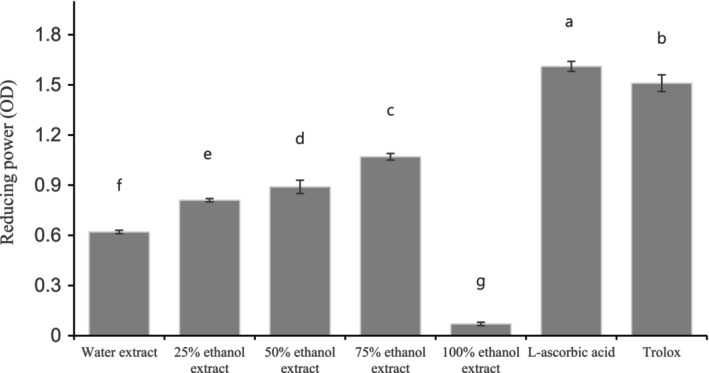
Reducing power of perilla seed meal of ethanol extract with different concentrations. Means followed by different letters (a–g) within different solvents are significantly different (*p* < .05).

Duh et al. (1999) reported the relationship between antioxidant activity and reducing power, where the higher reducing power of antioxidants often has a strong ability to donate electrons. Therefore, reducing power is normally used to forecast the antioxidant capacity. The reducing power of the PSME with L‐ascorbic acid and Trolox as reference standards revealed that the concentrations of the PSME had dependent effects and that the reducing power value of the 75EE was 1.07 OD at 0.5 μg/mL (*p* < .001; Figure 2). The reducing power value increased from the WE to the 75EE and drastically decreased for the 100% EE. Furthermore, the reducing power of the 75EE was very close to that of L‐ascorbic acid and Trolox. The results explain that the 75EE can transform free radicals into more stable products and perform as a good electron and hydrogen donor. Singh et al. (2021) observed a similar pattern of increased reducing power activity in a 75% ethanol extract of *Hibiscus sabdariffa* and decreased reducing power in the 50% and 100% ethanol extracts.

### 3.5 α‐Amylase and α‐glucosidase inhibition

**FIGURE 3:**
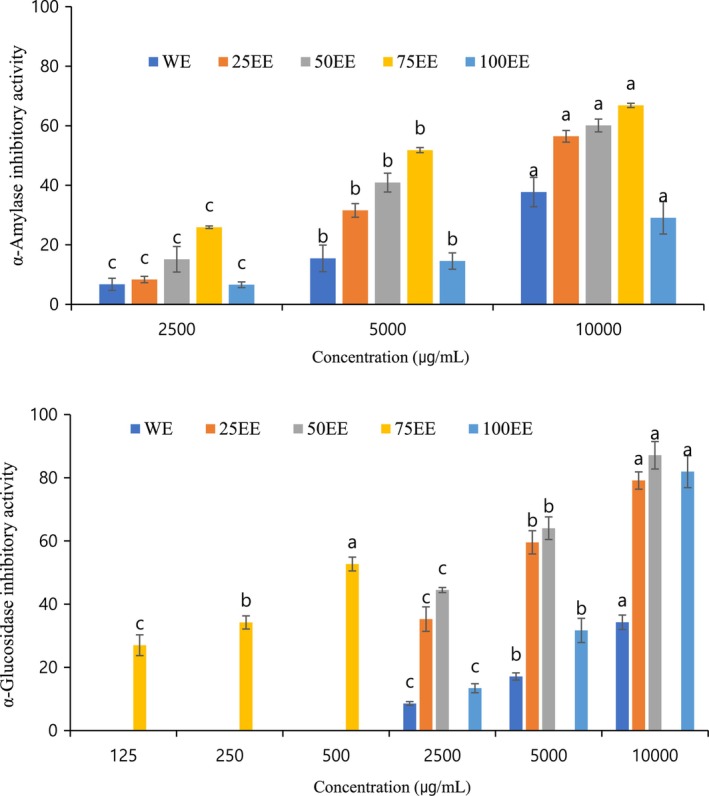
α‐Amylase and α‐glucosidase inhibitory activity of perilla seed meal of ethanol extract with different concentrations, water extract (WE), 25% ethanol extract (25EE), 50% ethanol extract (50EE), 75% ethanol extract (75EE), and 100% ethanol extract (100EE). Values with different letters (a–c) in different concentrations are significantly different (*p* < .05).

α‐Amylase and α‐glucosidase perform vital roles in carbohydrate digestion. Dietary carbohydrates are degraded into disaccharides depending on α‐amylase (Satoh et al., 2015). In the small intestine, disaccharides (such as maltose and sucrose) are degraded into monosaccharides (such as glucose) by α‐glucosidase prior to absorption (Fatmawati et al., 2011; Oboh et al., 2009). Inhibition of α‐amylase and α‐glucosidase can decrease the rate of starch digestion, slowing down the increase in postprandial blood glucose content. Therefore, α‐amylase and α‐glucosidase are considered therapeutic targets for postprandial hyperglycemia modulation in Type 2 diabetes mellitus. The inhibitory effects of α‐amylase and α‐glucosidase increased with increasing ethanol concentrations from 25% to 75%, but the inhibitory effect of α‐glucosidase significantly decreased for the 100EE (*p* < .001). Regarding the IC_50_ values (Table 3), the 75EE exhibited the greatest activity based on its potent inhibitory effect against α‐amylase and α‐glucosidase, with IC_50_ values of 5196.62 ± 248.38 and 361.28 ± 2.01, respectively. In the present study, the inhibitory effect of α‐glucosidase on the 75EE was very close to that of acarbose. The percentage inhibitions of the 75EE against α‐amylase and α‐glucosidase were significantly higher than those of the other extracts (Figure 3). α‐Glucosidase inhibition activity, the IC_50_ of methanol extract from sesame seed meal was 375 μg/mL (Reshma et al., 2013). The discarded seed can be used potentially for antidiabetic activity.

**TABLE 3:** α‐Amylase and α‐glucosidase inhibitory activity of perilla seed meal of ethanol extract with different concentrations.

Solvents	α‐Amylase inhibitory activity IC_50_ (μg/mL)	α‐Glucosidase inhibitory activity IC_50_ (μg/ml)
Water extract	>10,000	>10,000
25% ethanol extract	8405.59 ± 23.90^a^	4398.23 ± 144.87^b^
50% ethanol extract	7956.40 ± 134.27^b^	3211.47 ± 255.96^c^
75% ethanol extract	5196.62 ± 248.38^c^	5196.62 ± 248.38^c^
100% ethanol extract	>10,000	6239.82 ± 92.93^a^
Acarbose	215.71 ± 5.82^d^	184.90 ± 23.23^d^

*Note*: Means followed by different letters (a–d) within the same column are significantly different (*p* < .05). All values were expressed as mean ± SD (*n* = 3). The IC_50_ values are defined as the extract concentration to inhibit 50% of enzyme activity.

### 3.6 LC–MS/MS analysis

The purpose of the liquid chromatography‐electrospray ionization‐mass spectrometry analysis was to increase the nutritional potential and value of the perilla seed meal. The evaluation of the phenolic compounds was performed by mass spectra and the data were compared with reference compounds and the literature (Guan et al., 2014; Paradee et al., 2019; Zhang et al., 2018; Zhou et al., 2014). Table 4 explains the identified peaks, retention times, and pseudomolecular ions, as well as the concentration of each phenolic component discovered in the by‐product of perilla seed. Five phenolic compounds were discovered in the ethanolic extract: four phenolic acids (rosmarinic acid, benzoic acid, caffeic acid, and vanillic acid) and one flavonoid (apigenin). For example, the retention time of compound 1 was identified as 10.81 min based on the presence of a main peak at *m*/*z* 117.125. The retention times of compounds 2, 3, 4, and 5 were determined to be 9.18, 10.24, 8.29, and 8.42 min, respectively, and assigned according to the presence of main peaks at m/z 161.018, 77.27, 135.08, and 152.02, respectively. The amount of individual phenolic compounds, as summarized in Table 3, showed an important difference between the concentrations of the ethanol extracts.

**TABLE 4:** Phenolic compounds detected in perilla seed meal of ethanol extract with different concentrations.

No	Compounds	Retention time (min)	Molecular mass	[M‐H]− m/z	Water extract	25% ethanol extract	50% ethanol extract	75% ethanol extract	100% ethanol extract
1	Caffeic acid	8.29	179	135.08	1.586 ± 0.05^a^	1.059 ± 0.03^b^	0.726 ± 0.01^e^	0.918 ± 0.02^c^	0.803 ± 0.05^d^
2	Vanillic acid	8.42	167	152.02	0.333 ± 0.00^c^	0.266 ± 0.00^d^	0.234 ± 0.01^e^	0.433 ± 0.01^b^	0.909 ± 0.01^a^
3	Rosmarinic acid	9.18	359	161.018	35,229.016 ± 2340.23^a^	30,502.170 ± 1672.16^b^	28,628.337 ± 1604.31^b^	25,132.752 ± 1644.87^c^	1583.593 ± 112.53^d^
4	Benzoic acid	10.24	121	77.27	0.378 ± 0.01^d^	2.201 ± 0.03^c^	2.998 ± 0.06^b^	8.484 ± 0.03^a^	0.384 ± 0.03^d^
5	Apigenin	10.81	269	117.125	2.961 ± 0.04^d^	43.879 ± 0.27^c^	110.874 ± 1.99^b^	147.678 ± 4.98^a^	42.030 ± 1.01^c^

*Note*: Means followed by different letters (a–e) within the same column are significantly different (*p* < .05). All values were expressed as mean ± SD (*n* = 3). Concentration expressed as mg/100 g of DW (dry weight).

**FIGURE 4:**
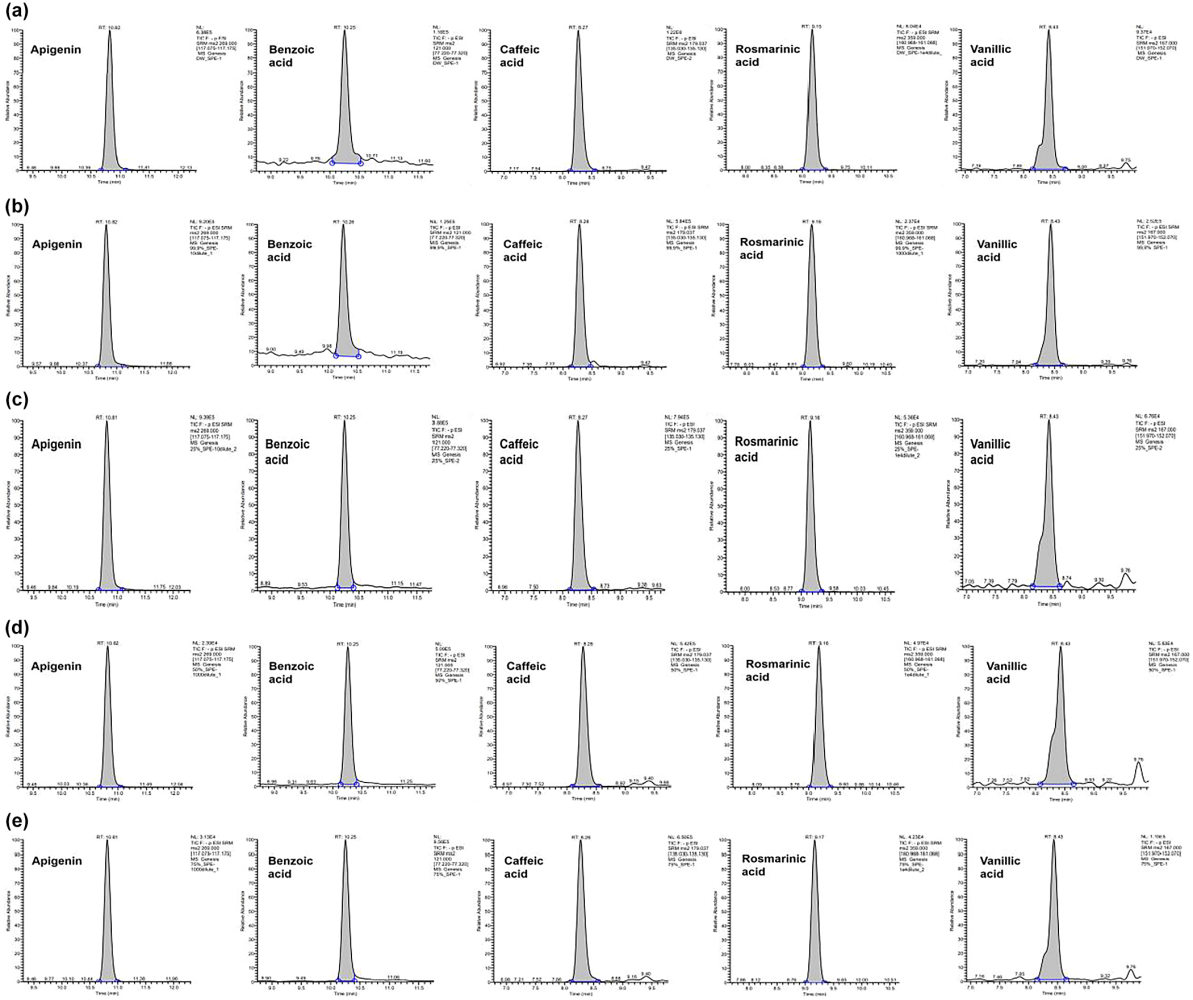
LC–MS/MS chromatograms of phenolic composition of perilla seed meal of ethanol extract with different concentrations, water extract (a), 25% ethanol extract (b), 50% ethanol extract (c), 75% ethanol extract (d), and 100% ethanol extract (e).

In this study, we reported for the first time the phenolic phytochemical profile analysis of PSME (Figure 4). Our results showed that there were significant differences attributed to the concentrations of the ethanol extracts (*p* < .05). Table 4 revealed that the major components of the samples were identified in the following order: rosmarinic acid > apigenin > caffeic acid > benzoic acid > vanillic acid. The rosmarinic acid content of the WE, 25EE, 50EE, 75EE, and 100EE was 35,229.016 ± 2340.23, 30,502.170 ± 1672.16, 28,628.337 ± 1604.31, 25,132.752 ± 1644.87, and 1583.593 ± 112.53 mg/100 g, respectively (*p* < .001). Yan and Zheng (2017) revealed that the major soluble phenolic component in perilla seed flour was rosmarinic acid, which accounted for about 73% of all phenolic compounds. The rosmarinic acid isolated from PSM exhibited the highest DPPH radical‐scavenging activity, which increased in a concentration‐dependent manner and had significant positive correlations with antioxidant activities (Zhou et al., 2014). Similarly, Gai et al. (2017) reported that the strong antioxidant activity of the *Perilla* plant was associated with its high rosmarinic acid content. The concentrations of the relative phenolic compounds differed by each ethanol concentration. Chumphukam et al. (2018) reported that the rosmarinic acid in the ethanol extracts of the perilla seed and seed meal were 158.8 and 141.2 μg/mg, respectively. According to López‐Fernández et al. (2020), the phenolic compound solubility is influenced by the chemical composition of the matrix constituents and the polarity of the extraction solvent. The chemical profile and extraction of phenolics from any plant material depend on the solvent used (Dai & Mumper, 2010). As a result, extraction with higher polarity solvents is required to increase the overall polarity of the solvent mixture (Stalikas, 2007).

## 4 CONCLUSION

This study evaluated the total phenolic and total flavonoid contents and investigated the potential antioxidant by‐product of *Perilla frutescens* seed extracted with ethanol of different concentrations (water, 25%, 50%, 75%, and 100%), while also focusing on the inhibitory effects of α‐amylase and α‐glucosidase. The results suggested that 75EE may be an excellent extraction solvent for extracting chemical components from perilla seed meal. In addition, 75EE could be used as an antioxidant, which contains high phenolic and flavonoid compound concentrations. Moreover, analysis of the phenolic compounds in perilla seed meal extracts by LC–MS/MS indicated the presence of apigenin, rosmarinic acid, benzoic acid, caffeic acid, and vanillic acid. The 75EE had better potential to inhibit α‐amylase and α‐glucosidase. Overall, this study offered essential scientific support to the application of *P. frutescens* seed by‐products as nutraceuticals for the dietary management of oxidation and diabetes.

## AUTHOR CONTRIBUTIONS

**Gayoung Choi:** Writing – original draft (equal). **Youngsil Han:** Project administration (equal). **Kihyeon Sim:** Project administration (equal). **Myunghyun Kim:** Writing – review and editing (equal).

## CONFLICT OF INTEREST STATEMENT

The authors confirm that they have no conflicts of interest with respect to the work described in this manuscript.

## ETHICS STATEMENT

This article does not contain any studies performed with human participants or animals by any of the authors.

## CONSENT TO PARTICIPATE

Corresponding and all the coauthors are willing to participate in this manuscript.

## CONSENT FOR PUBLICATION

All authors are willing for publication of this manuscript.

## Data Availability

Research data are not shared.
